# The importance of cardiovascular pathology contributing to maternal death: Confidential Enquiry into Maternal Deaths in South Africa, 2011–2013

**DOI:** 10.5830/CVJA-2016-008

**Published:** 2016

**Authors:** Priya Soma-Pillay, Joseph Seabe, Priya Soma-Pillay, Joseph Seabe, Karen Sliwa

**Affiliations:** Department of Obstetrics and Gynaecology, Maternal and Foetal Medicine, University of Pretoria and Steve Biko Academic Hospital, Pretoria, South Africa; Department of Obstetrics and Gynaecology, Tembisa Hospital, Tembisa, South Africa; National Committee for the Confidential Enquiry into Maternal Deaths, South Africa; National Committee for the Confidential Enquiry into Maternal Deaths, South Africa; Hatter Institute for Cardiovascular Research in Africa, and IDM, Department of Medicine, Faculty of Health Sciences, University of Cape Town, South Africa; Soweto Cardiovascular Research Unit, University of the Witwatersrand, Johannesburg; Inter-Cape Heart Group, Medical Research Council South Africa, Cape Town, South Africa

**Keywords:** cardiac disease in pregnancy, valve disease, valve thrombosis, rheumatic heart disease, cardiomyopathy, peripartum cardiomyopathy

## Abstract

**Aims:**

Cardiac disease is emerging as an important contributor to maternal deaths in both lower-to-middle and higher-income countries. There has been a steady increase in the overall institutional maternal mortality rate in South Africa over the last decade. The objectives of this study were to determine the cardiovascular causes and contributing factors of maternal death in South Africa, and identify avoidable factors, and thus improve the quality of care provided.

**Methods:**

Data collected via the South African National Confidential Enquiry into Maternal Deaths (NCCEMD) for the period 2011–2013 for cardiovascular disease (CVD) reported as the primary pathology was analysed. Only data for maternal deaths within 42 days post-delivery were recorded, as per statutory requirement. One hundred and sixty-nine cases were reported for this period, with 118 complete hospital case files available for assessment and data analysis.

**Results:**

Peripartum cardiomyopathy (PPCM) (34%) and complications of rheumatic heart disease (RHD) (25.3%) were the most important causes of maternal death. Hypertensive disorders of pregnancy, HIV disease infection and anaemia were important contributing factors identified in women who died of peripartum cardiomyopathy. Mitral stenosis was the most important contributor to death in RHD cases. Of children born alive, 71.8% were born preterm and 64.5% had low birth weight. Seventy-eight per cent of patients received antenatal care, however only 33.7% had a specialist as an antenatal care provider. Avoidable factors contributing to death included delay in patients seeking help (41.5%), lack of expertise of medical staff managing the case (29.7%), delay in referral to the appropriate level of care (26.3%), and delay in appropriate action (36.4%).

**Conclusion:**

The pattern of CVD contributing to maternal death in South Africa was dominated by PPCM and complications of RHD, which could, to a large extent, have been avoided. It is likely that there were many CVD deaths that were not reported, such as late maternal mortality (up to one year postpartum). Infrastructural changes, use of appropriate referral algorithm and training of primary, secondary and tertiary staff in CVD complicating pregnancy is likely to improve the outcome. The use of simple screening equipment and point-of-care testing for early-onset heart failure should be explored via research projects.

## Aims

Cardiac disease is emerging as an important indirect cause of maternal death globally. Cardiac conditions may be pre-existing, such as rheumatic heart disease (RHD) or congenital heart disease and may be unmasked by the increased haemodynamic load in pregnancy, or may be caused by the pregnancy, for example hypertensive disorders or peripartum cardiomyopathy (PPCM).^1^

Compared with child mortality, maternal mortality has been more difficult to track over time at a national level, in particular in middle- to lower-income countries (LMICs). Major challenges include incomplete data sets, inexperience of the physicians in applying the classifications, misclassification of maternal deaths to other causes in countries with complete vital registration and medical certification of causes of deaths.^2^ However, many cases remain unreported due to lack of linkage to the causality of the pregnancy.

Maternal death is rarely reported beyond six weeks postpartum. The ICD 10 classification (version 10) defining late maternal death (six weeks to one year) is often not applied. This leads to the fact that death due to, for example PPCM, which often only presents three to five months postpartum, death due to left ventricular dysfunction and heart failure related to hypertensive disorders in pregnancy, or death related to right heart failure in complex congenital heart disease remains unreported and, therefore, not adequately addressed. There is a profound lack of knowledge on cardiac disease contributing to morbidity and mortality, which impacts on foetal outcome, not only in South Africa but on a global level.

The objectives of the study were to determine the cardiovascular causes and contributing co-morbidities of maternal death in South Africa, and to identify avoidable factors and missed opportunities. The goal is to develop strategies to improve quality of care, with the ultimate aim to reduce maternal death due to cardiovascular disease.

## Methods

This study was an audit of maternal deaths due to cardiovascular disease in South Africa for the period 2011–2013. Maternal death is defined as the death of a woman while pregnant, or within 42 days of termination of pregnancy, irrespective of the duration and site of pregnancy, from any cause related to or aggravated by the pregnancy or its management, but not from accidental or incidental causes.^3^

**Fig. 1.**
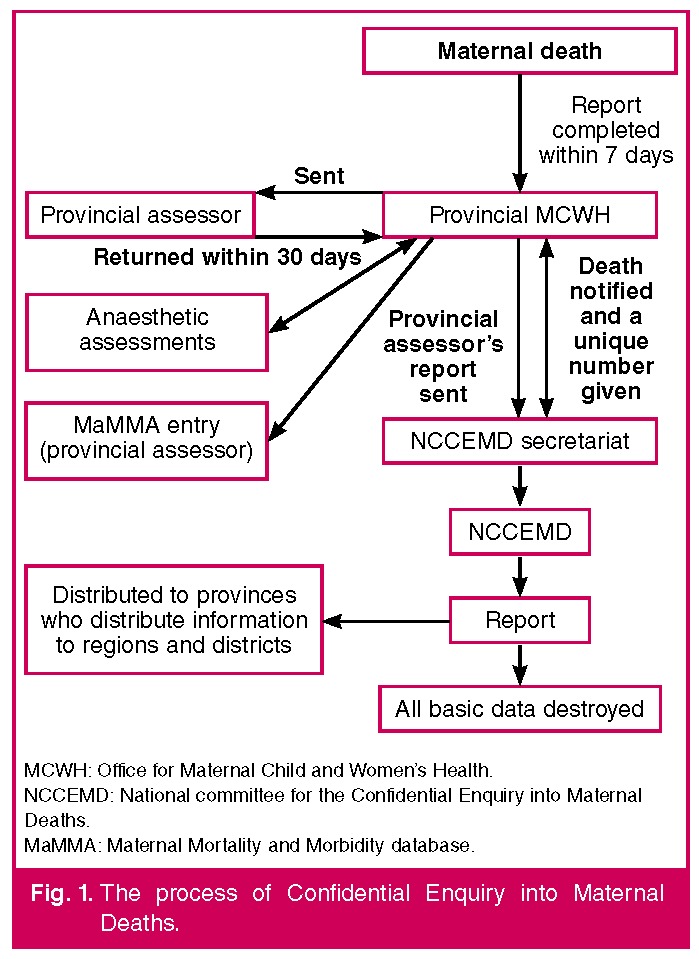
The process of Confidential Enquiry into Maternal Deaths.

In South Africa it is currently not a statutory requirement to document and record late maternal deaths (up to one year postpartum, ICD 10 code, version 10). Maternal deaths are notifiable by law in South Africa. Following the death of a mother, it is the responsibility of the clinician caring for the mother to fill in the Maternal Death Notification form (MDNF). This form, together with a copy of the patient’s clinical notes, must be sent to the Provincial Maternal Child and Woman’s Health Office within seven days of the maternal death. (Fig. 1) describes the process of the Confidential Enquiry into Maternal Deaths.^4^

One hundred and sixty-nine cases of maternal deaths related to cardiac disease were reported to the National Committee for the Confidential Enquiries into Maternal Deaths (NCCEMD) and entered on the MaMMA’s database for the triennium 2011–2013. One hundred and eighteen hospital case files with complete data were available for assessment, data extraction and analysis. Permission was obtained from the NCCEMD and the Department of Health of South Africa for this audit to be conducted and presented.

## Results

## Overall demographic data, antenatal risk factors and mode of delivery

The demographic information of the study population is shown in Table 1. The majority of the women were black African, with a mean age of 28.6 years and a parity of less than 2. More than one-third of the patients were HIV positive. Most patients had a low systolic blood pressure of 116 ± 28.6 mmHg and an elevated heart rate (HR).

**Table 1:** Demographic data of the study population (n = 118)

*Parameters*	Demographic data
Race	
African, n (%)	104 (88.2)
Coloured, n (%)	7 (6.9)
White, n (%)	5 (3.9)
Indian, n (%)	2 (1.7)
Age (years)	
Mean (± SD)	28.6 (6.49)
Range	17–43
Obstetric history	
Parity median (range)	1 (1–6)
Gravidity median (range)	2 (1–6)
HIV disease status, n (%)	
HIV positive	50 (42.4)
HIV negative	56 (47.5)
Unknown disease status	12 (10.2)
CD4 count median (SD)	275 (18-839)
Haemoglobin at presentation	
Haemoglobin (g/dl), mean (± SD)	9.5 (1.8)
Range	5–12
Heart rate at presentation	
Heart rate (bpm), mean (± SD)	115 (25.7)
Range	69–180
Blood pressure at presentation	
Systolic blood pressure (mmHg), mean (± SD)	116.3 (28.6)
Diastolic blood pressure (mmHg), mean (± SD)	65.1 (20.7)

Table 2 summarises the antenatal risk factors (as reported in the MDNF) as documented at the first antenatal visit of the pregnant mother. Some patients had more than one risk factor.

**Table 2:** Antenatal risk factors (n = 118)

*Risk factor*	*Number (%)*
With known heart disease	46 (39.7)
Smoking (past and current)	11 (9.3)
Tuberculosis (past and current)	8 (6.8)
Hypertension	43 (36.4)
Proteinuria in current pregnancy	22 (18.6)
Glycosuria in current pregnancy	12 (10.2)
Anaemia (haemoglobin < 10 g/l)	30 (26.8)

**Fig. 2.**
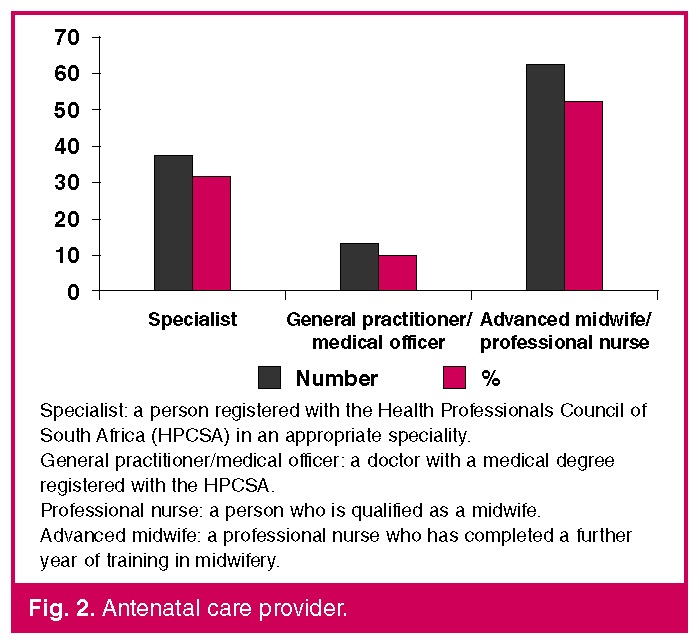
Antenatal care provider.

Ninety-two (78%) patients attended antenatal clinics, but only 44.3% of patients booked for antenatal care before 20 weeks’ gestation. (Fig. 2) describes the level of antenatal care received by the women who died. Forty (33.7%) mothers delivered vaginally, 31 (26.3%) by Caesarean section, and 47 (39.8%) mothers were undelivered. The average gestation at delivery was 32 weeks.

Fifty-one (71.8%) babies were born preterm (< 37 weeks’ gestation). The average birth weight of babies born alive was 2 558 g. Of the babies born alive, 37 (64.5%) were low birthweight (< 2.5 kg) babies.

## Cardiovascular conditions and co-morbidities leading to death

An electrocardiogram and echocardiogram was performed in only 42.9% (*n* = 50) and 34% (*n* = 40) of patients, respectively. The mean heart rate of the patients who died was 115 beats per minute (Table 1); 19 were hypertensive (systolic BP > 140 mmHg) and eight were hypotensive (systolic BP < 100 mmHg).

The majority of women (69%, *n* = 71) died after delivery, while the remaining 47 (31%) died during the antenatal period. For the mothers who died in the postpartum period, death occurred 11 ± 10.7 days postpartum. Seventy-two per cent of mothers presented to the health institutions in a critically ill condition, while 6% of the mothers were dead on arrival. The maternal deaths occurred at the following health localities: community health clinics, five patients (4.12%); level one hospital, 25 (21.7%); level two hospitals, 34 (28.9%); level three hospitals, 50 (42.3%) and private hospitals, four (3.1%).

**Fig. 3.**
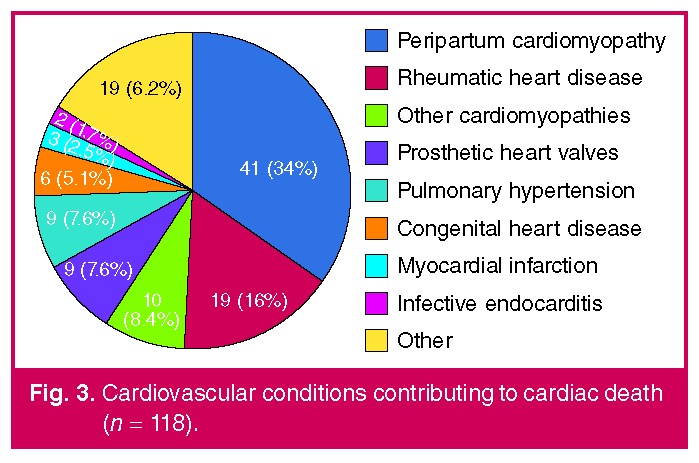
Cardiovascular conditions contributing to cardiac death (n = 118).

The diagnosis contributing to cardiac death is illustrated in (Fig. 3). PPCM (34%) and complications of RHD, which includes un-operated cases, as well as cases with prosthetic valve disease (25.3%), were the most important diagnoses leading to maternal death.

## PPCM and other cardiomyopathy

There were 41 deaths due to PPCM. All cases were newly diagnosed as none of the maternal records documented a previous history of cardiomyopathy. Twelve (29.3%) deaths occurred at level three institutions, 14 (34.2%) at level two facilities and 15 (36.6%) at level one or community health clinics. Twenty (48.8%) mothers presented with acute symptoms in the postpartum period. Death occurred in nine (22.0%) patients who were undelivered, and 32 (78.1%) were postpartum.

The most important antenatal co-morbidities identified among the women who died due to a cardiomyopathy were: hypertension, 22 patients (53.7%), HIV infection, 17 (41.5%) and anaemia, 15 (36.6%). Twenty (48.8%) mothers however had a haemoglobin level of < 10 g/dl when they presented in acute cardiac failure. In most cases, a clinical diagnosis was made, in only 12 (30%) cases was an electrocardiogram performed, and an echocardiogram was done in five (13%) cases to confirm diagnosis of a cardiomyopathy.

## Rheumatic heart disease

There were 35 maternal death files due to complications of RHD available for assessment. There were 19 cases of valvular heart disease, four deaths due to complications of prosthetic heart valves (presumed to be rheumatic in origin in this South African population), two deaths due to infective endocarditis and five cases of underlying valvular lesions complicated by pulmonary hypertension. Mitral stenosis was the most common valvular lesion contributing to maternal death (> 50% of cases with valvular lesions), followed by severe tricuspid incompetence (*n* = 4), mixed mitral valve disease (*n* = 2), aortic stenosis (*n* = 2), and one case of isolated severe mitral regurgitation.

All four patients with mechanical heart valve prostheses died due to valve thrombosis. Two patients were non-compliant with anti-coagulant medications. One patient was treated with low-molecular weight heparin without any monitoring of anti-Xa levels and no anti-coagulant was prescribed in the other patient post-delivery.

The average age of the mothers who died was 28.1 ± 6.49 years. Twenty-four (68.5%) mothers presented for antenatal care with a known history of cardiac disease, while 11 (31.5%) mothers had undiagnosed cardiac lesions prior to pregnancy. Twenty-six (74.1%) mothers booked for antenatal care but only 12 (34. 3%) were managed at a tertiary institution during the antenatal period. Death occurred in the following institutions: level one facility, two patients (5.7%); level two hospitals, 11 (3.4%), and level three hospitals, 22 (62.9%).

Table 3 summarises the factors contributing to death for the entire study population, as well as women with PPCM and RHD. This information was obtained from the MDNF and is the opinion of the clinician reporting the death. Some patients had more than one avoidable factor.

**Table 3:** Factors contributing to death for the two major disease groups

*Avoidable factor*	*Whole group n (%)*	*Peripartum cardiomyopathy n (%)*	*Rheumatic heart disease n (%)*
Patient delay in seeking help	49 (41.5)	16 (39.0)	16 (45.7)
Lack of expertise by medical staff managing case	35 (29.7)	16 (39.0)	12 (34.3)
Delay in referral to appropriate level of care	31 (26.3)	13 (31.7)	8 (22.9)
Delay in appropriate action	43 (36.4)	15 (36.6)	15 (42.9)

In 24.3% of cases, the assessors believed that different management could reasonably have been expected to affect outcome. The problems of failure to make a diagnosis, incorrect management and delay in referring patients to the appropriate level of care were important factors that contributed to cardiac mortality (Table 4).

**Table 4:** Foetal outcome for all pregnant women (n = 118), women with peripartum cardiomyopathy (n = 41) and rheumatic heart disease (n = 35)

**	*Whole group n (%)*	*Peripartum cardiomyopathy n (%)*	*Rheumatic heart disease n (%)*
*In utero* death	11 (9.3)	4 (9.8)	3 (8.6)
Gestation, mean (± SD)	32 (7.7)	35.6 (6.9)	27.2 (8.9)
Born preterm (< 37 weeks gestation)	51 (71.8)	14 (34.1)	15 (42.9)
Low birth weight (< 2 500 g)	37 (64.5)	10 (24.3)	11 (31.4)

## Discussion

This study has shown a disease pattern markedly different to that seen in high-income countries, with cardiomyopathies and RHD most commonly leading to death, often complicated by HIV/AIDS, hypertension and anaemia as co-morbidities. Confidential inquiries on maternal death reports from European high-income countries and the European EURObservational Research Programme registry on cardiac disease in pregnancy typically report operated congenital heart disease as the most common mode of death.^5^

## Access to care, avoidable factors and late maternal death

The majority of patients attended antenatal care but booked late. Only one-third had access to a specialist as an antenatal care provider. The most important avoidable factors contributing to death included: delay in patients seeking help (> 50% of patients), lack of expertise of medical staff managing the case (30%), delay in referral to the appropriate level of care, and inappropriate action.

A recent single-centre prospective cohort study from Groote Schuur Hospital^6^ has reported that most deaths were due to different forms of cardiomyopathies, with only two related to complications attributable to sepsis and thrombosis affecting prosthetic heart valves. However, eight out of the nine deaths reported in this 152-patient cohort with a six-month post-delivery outcome period would not have been reported if the definition of death within 42 days had been applied. This highlights the underestimation of the number of cardiac deaths related to pregnancy as a result of the late presentation, and these deaths are especially important among women with familial or PPCM.

The European Society of Cardiology working group on PPCM has defined PPCM as an ‘idiopathic’ cardiomyopathy presenting with heart failure secondary to left ventricular systolic dysfunction towards the end of pregnancy, or in the months following delivery, where no other cause of heart failure is found.^7^ Patients most commonly present two to three months postpartum and therefore outside the 42 days reporting period.^8^ This condition may be difficult to distinguish from other forms of cardiomyopathy, such as familial or pre-existing idiopathic dilated cardiomyopathy, which usually presents prior to pregnancy or in the second or third trimester.

Reported incidence for PPCM varies among different geographic regions, with potential hotspots in Africa (1:100 to 1:1 000).^9^ There has been an increase in the reporting of PPCM in high-income countries in the past decade and this is probably due to increasing awareness created by a large prospective international registry on PPCM, the ESC EURObservational Research Programme (http://www.eorp.org).^10^ At present the overall mortality rate is between 10 and 25%.

The fact that more than two-thirds of all deaths occurred post-partum and that PPCM was the most common condition leading to death in this tri-annual report is an important finding. It also implies that the maternal death rate in South Africa, which is already estimated to be 176/100 000,^2^ is underestimated, as death could only be reported until 42 days postpartum. Cardiomyopathies or other causes of left ventricular dysfunction that often present with heart failure or severe arrhythmia leading to death beyond that period is of major concern. These deaths are pregnancy related, even at late presentation.

Interventions to prevent these deaths include adequate counselling about the risks of future pregnancy, access to adequate contraceptive services, termination of breastfeeding, and use of the medication bromocriptine^8^ in patients with PPCM. This is crucial as available data strongly suggest that subsequent pregnancy in patients with PPCM is associated with a high risk of relapse and death.^11^

## Improving care for women with undiagnosed, diagnosed and operated RHD

Valvular heart disease in pregnant women, whether due to congenital or acquired aetiologies, such as RHD, poses a challenge to clinicians and their patients. Significant valve disease increases the risk of pregnancy to the mother and foetus and requires a careful preconception risk assessment and, subsequently during pregnancy, specialised care to minimise maternal and foetal morbidity and mortality.

All women with valvular heart disease should ideally have preconception evaluation, including advice on risk prediction and contraception by a joint cardiac–obstetric team. Zühlke and co-authors reported recently from the REMEDY study that among 1 825 women of child-bearing age with RHD, only 3.6% were on contraception.^12^ A recent publication by Sliwa *et al.*^13^ summarises how counselling on maternal and offspring risk should be carried out in women with valvular heart disease, according to the modified World Health Organisation (WHO) classification, and should include information on complications such as heart failure and valve thrombosis, which can occur during and beyond the immediate delivery period.

Management of the patients in our cohort was clearly sub-optimal. Many patients presented late to healthcare providers and this was possibly due to lack of knowledge of the underlying cardiac problem. This could potentially be improved by providing better information by a counsellor, cell phone/ web-based information or via short featured video clips, e.g. www.heduafrica.org and MomConnect website (www.rmch.org/wp-content/uploads/2014/08/MomConnect-Booklet.pdf). Appropriate guidance in referral to secondary and tertiary care hospitals with dedicated cardiac disease in maternity clinics should be implemented and is currently being explored in South Africa.

## Cardiac disease contributing to institutional maternal mortality rate in South Africa

There has been a steady increase in the institutional maternal mortality rate (iMMR) for cardiac disease over the last decade in South Africa.^14^ The iMMR for cardiac disease in 2005–2007 was 3.73 and this increased to 5.64 during 2008–2010, and to 6.00 per 100 000 during 2011–2013. After non-pregnancy-related infections, cardiac disease is the second most common cause of indirect maternal death.

The Saving Mothers reports of 2002–2004 and 2005–2007 have grouped all cases of cardiomyopathy (peripartum cardiomyopathy and other cardiomyopathies) in one category when analysing causes of cardiac death.^4^,^15^ In these reports, complications of RHD and cardiomyopathy were the most important and equal contributors to cardiac deaths. In the triennium 2011–2013, the number of deaths due to peripartum cardiomyopathy was more than double that of complications related to RHD, and formed 34% of the total number of cardiac deaths.

Our data suggest that care in the postpartum period needs to be improved, possibly including earlier referral to the general cardiac clinic or cardiomyopathy clinic. However, joint obstetric–medical–cardiac clinics would be the optimal approach for these patients. Medical physicians and cardiologists need to be actively involved in the postpartum care of women with cardiac disease. A need to provide focused training to medical registrars has already been identified. Most tertiary level hospitals in South Africa, such as Steve Biko Academic Hospital, Pretoria and Groote Schuur Hospital, Cape Town, now provide a bi-weekly cardiac–obstetric clinics and regular obstetric medicine lectures in their registrar training programmes.

The use of simple screening equipment, such as hand-held echocardiography and point-of-care testing for early-onset heart failure, should be explored via research projects. A recent publication evaluated the ability of medical students who had previously received training in echocardiography (eight hours) to detect RHD. The students’ averaged sensitivity for diagnosing RHD was 81%, while specificity was 95%.^16^ Handheld echocardiography as a routine diagnostic facility should be considered as a training module for students and could improve detection of significant cardiac disease in primary and secondary care.^17^

Research on the use of simple point-of-care testing on NT-proBNP, a marker of early heart failure, could lead to earlier detection of heart failure related to various forms of CVD.

## Limitations

The retrospective analysis of patients’ files, and the limited number of investigations that can be performed in primary or secondary care or due to the emergency condition itself clearly impacts on the quality of data that can be collected via such a retrospective audit. It is a major limitation that not all patients’ files were accessible. In many cases the diagnosis, often made by a junior doctor, could not be verified or the patient died prior to reaching a higher-level hospital.

## Conclusion

The pattern of CVD contributing to maternal deaths in South Africa was dominated by cardiomyopathies and complications of RHD, which could have been avoided to a large extent. There is most likely an underestimation of maternity-related death, as late maternal mortality (up to one year postpartum) is not recorded. Infrastructural changes, use of an appropriate referral algorithm and training of primary, secondary and tertiary staff in cardiovascular disease complicating pregnancy is likely to improve the outcome.
